# Prognostic and Predictive Significance of Primary Tumor Localization and HER2 Expression in the Treatment of Patients with KRAS Wild-Type Metastatic Colorectal Cancer: Single-Centre Experience from Serbia

**DOI:** 10.3390/jpm14080879

**Published:** 2024-08-20

**Authors:** Jelena Radić, Ivan Nikolić, Ivana Kolarov-Bjelobrk, Tijana Vasiljević, Aleksandar Djurić, Vladimir Vidović, Bojana Kožik

**Affiliations:** 1 Faculty of Medicine, University of Novi Sad, 21000 Novi Sad, Serbia; jelena.radic@mf.uns.ac.rs (J.R.); ivan.nikolic@mf.uns.ac.rs (I.N.); ivana.kolarov-bjelobrk@mf.uns.ac.rs (I.K.-B.); tijana.vasiljevic@mf.uns.ac.rs (T.V.); 2 Department of Medical Oncology, Oncology Institute of Vojvodina, 21204 Sremska Kamenica, Serbia; aleksandardjuric555@gmail.com (A.D.); vladimir_vidovic@hotmail.com (V.V.); 3 Department of Pathology and Laboratory Diagnostic, Oncology Institute of Vojvodina, 21204 Sremska Kamenica, Serbia; 4 Laboratory for Radiobiology and Molecular Genetics, Vinča Institute of Nuclear Sciences, National Institute of Republic of Serbia, University of Belgrade, 11000 Belgrade, Serbia

**Keywords:** metastatic colorectal cancer, primary tumor localization, anti-EGFR therapy, KRAS status, HER2 expression

## Abstract

The treatment of patients with metastatic colorectal cancer (mCRC) is complex and is impacted by the location of the primary tumor (LPT). Our study aims to emphasize the importance of LPT as a prognostic and predictive marker as well as to examine the significance of HER2 overexpression in patients with mCRC, particularly in relation to the response to Epidermal Growth Factor Receptor Antibody treatment (anti-EGFR therapy). In this study, 181 patients with Kirsten RAS (KRAS) wild-type mCRC who received anti-EGFR therapy were included. Among them, 101 had left colon cancer (LCC) and 80 had right colon cancer (RCC). Results demonstrated that patients with KRAS wild-type LCC had better median overall survival (OS) (43 vs. 33 months, *p* = 0.005) and progression-free survival (PFS) (6 vs. 3 months, *p* < 0.001) compared to those with RCC. Multivariate analysis identified mucinous adenocarcinoma (*p* < 0.001), RCC location (*p* = 0.022), perineural invasion (*p* = 0.034), and tumors at the resection margin (*p* = 0.001) as independent predictors of OS, while mucinous adenocarcinoma (*p* = 0.001) and RCC location (*p* = 0.004) independently correlated with significantly shorter PFS. In addition, human epidermal growth factor receptor 2 (HER2) positive expression was significantly associated with worse PFS compared to HER2 negative results (*p* < 0.001). In conclusion, LPT is an important marker for predicting outcomes in the treatment of wild-type mCRC using anti-EGFR therapy, since patients with RCC have a statistically significantly shorter PFS and OS. Further investigation is needed to understand the role of HER2 overexpression in wild-type mCRC, as these patients also exhibit shorter survival.

## 1. Introduction

The localization of the primary tumor (LPT) is an important prognostic and predictive factor that helps to a great extent in individualizing and personalizing treatment of patients with metastatic colorectal cancer (mCRC) [1]. The exact boundary between the left and right colon is still not precisely defined. Most often, the point of separation is the lienal flexure, so the right colon is up to the lienal flexure (vermiform appendix, appendix, ascending part, transverse part of the colon) and the distal part belongs to the left colon (descending part of the colon, sigmoid part and rectum) [2]. Although considered a single organ, the colon has segments that are different from each other histologically, physiologically, and molecularly [3,4,5,6,7,8]. One of the possible reasons for these differences lies in the fact that the left and right colon originate from different layers of the endoderm; the right colon (vermiform appendix, appendix, ascending part, proximal two-thirds of the transverse part of the colon) originates from the anterior and the left (distal third of the transverse part, descending part of the colon, sigmoid part and rectum) comes from the posterior layer [9]. Recent studies have found that both left (LCC) and right colon (RCC) cancers behave differently regarding epidemiology, pathohistology, clinical presentation, molecular characteristics, and metastatic patterns [4,10,11,12,13,14,15,16,17,18]. These multifactorial differences, caused by the LPT, might eventually lead to prognostic variations between RCC and LCC.

For CRC, it is important to determine all of the RAS mutations including mutations in the Kirsten RAS (KRAS) gene at exon 2 (codon 12/13), exons 3 and 4, and neuroblastoma RAS (N-RAS) mutations at exons 2–4 [19]. About 40% of colon tumors have mutations in the KRAS proto-oncogene, which are associated with resistance to biological therapy, i.e., monoclonal antibodies directed against epidermal growth factor receptor (anti-EGFR ab). Patients with KRAS wild-type (wt) tumors may have NRAS mutations in 10% of cases, making them resistant to the anti-EGFR therapy [20]. However, there are patients who, despite the absence of KRAS and NRAS mutations, still do not respond to targeted therapy. One reason for this is the B-Raf proto-oncogene (BRAF) mutation that occurs in about 8–12% of cases in patients who have all RAS wild-type tumors. BRAF has both prognostic and predictive significance in mCRC. Detection of the BRAF mutation indicates an unfavorable prognosis and predicts a non-response to anti-EGFR therapy [21,22,23].

One of the potential predictive factors in CRC could be HER2 (human epidermal growth factor receptor 2) [24]. The expression of these receptors in CRC is present in about 5% of patients, but the prevalence is higher, 5–14%, in RAS/BRAF wild-type tumors [20]. Since 2011, there has been a discussion of a possible effect of overexpression of HER2 receptors on resistance to biological therapy with anti-EGFR antibodies. The role of the HER2 receptor as a prognostic factor is still unclear. Some authors associate overexpression of HER2 receptors with a worse prognosis, including shorter time to disease progression and shorter overall survival [25], although this association has not been confirmed in similar studies [26,27].

This study aims to examine the predictive and prognostic role of the LPT in the treatment of mCRC patients, since the ultimate goal of the treatment is to improve the effectiveness and specificity of anti-EGFR therapy. Our study indicates the significant importance of the LPT in the treatment of mCRC patients. It should be emphasized that this parameter does not require additional pathological, immunohistochemical, or molecular testing, and it is easily available to any medical oncologist. Also, given the existing variability of clinical outcomes and responses to the treatment of patients with mCRC, it is clear that there is a need to identify new molecular biomarkers for the most accurate personalization and individualization of the treatment. Although the HER2 amplification could predict a lack of response to anti-EGFR therapy in patients with mCRC, we also wanted to examine the importance of overexpression of HER2 in our cohort of wt mCRC patients.

## 2. Materials and Methods

### 2.1. Patients and Treatment

This observational study included a total of 181 patients with pathohistologically verified colon cancer in the period from January 2009 to July 2021, who at the time of diagnosis had a metastatic disease or developed metastases during the disease. Data on clinically pathological parameters were collected from the medical documentation of the Oncology Institute of Vojvodina for patients located in the electronic database. The study was conducted in accordance with the Declaration of Helsinki and approved by the Ethics Committee of the Oncology Institute of Vojvodina (protocol code 4/17/1-386272-20, date of approval 5 December 2017).

**Figure 1:**
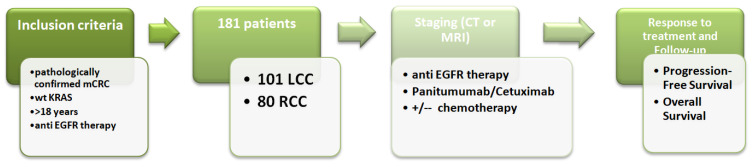
Study design and inclusion criteria for patients with KRAS wt mCRC.

Pathological parameters of the tumor, including pathohistological type of tumor, degree of differentiation, presence of vascular and perineural invasion (LVI, PNI), tumor infiltration lymphocytes (TIL), lymph node involvement, number of examined lymph nodes, positivity of resection margins, clinical course of the disease, progression-free survival (PFS), and overall survival (OS), were analysed in all patients. KRAS status was determined via the Cobas DNA Sample Preparation Kit and the Cobas z480 instrument, using a PCR method of DNA extraction from FFPE tumor tissue as part of the standard clinical procedure for metastatic CRC patients. Patients with mCRC KRAS wt tumors received anti-EGFR therapy (cetuximab—administered once every two weeks, dosed at 500 mg/m^2^ and panitumumab—administered once every two weeks, dosed at 6 mg/kg) in addition to the standard chemotherapy protocols administered once every two weeks (Folfox4—oxalipatin dosed at 80 mm/m^2^ + DeGramount; Folfiri—irinotecan dosed at 180 mg/m^2^ + DeGramount; DeGramount—leucovorin dosed at 400 mg/m^2^ via IV over 2 h on day 1, followed by Fluorouracil dosed at 400 mg/m^2^ via IV push on day 1 followed by Fluorouracil dosed ar 1200 mg/m^2^ via IV continuous infusion daily on days 1–2 [dosed at 2400 mg/m^2^ via IV over 46–48 h]). The patients’ responses to the applied therapy were monitored using radiological methods such as computed tomography scan (CTS) or magnetic resonance imaging (MRI) (Figure 1).

As an additional prognostic parameter for patients with mCRC, an immunohistochemical examination of tumor tissue for detecting the presence of the HER2 gene protein product expression was performed by using the DAKO Polyclonal Rabbit Anti-Human c-erbB2 oncoprotein. Determination of the intensity of the immunohistochemical reaction was performed according to the consensus recommendations of the panel of pathologists, i.e., the HERACLES diagnostic criteria from the study of the same name [28]. According to the HERACLES diagnostic criteria [28], positive expression results are recorded only in cases with intense circumferential, basolateral, or lateral response in more than 10% of cells.

### 2.2. Statistical Analysis

For statistical data processing, the software package Statistical Package for Social Sciences—SPSS 21 was used. Numerical features are represented by medians (arithmetic mean) and measures of variability (range of values, standard deviation), and attribute features are represented using frequencies and percentages. The comparison of the values of numerical features between the two groups was performed using the Student’s *t*-test, i.e., the non-parametric Mann–Whitney test. Testing the difference in the frequencies of attribute features was performed using the χ^2^ test. The examination of the connection between the two traits was performed using the Pearson correlation coefficient. In order to analyse the time to disease progression as well as mortality, the Kaplan–Meier assessment of survival function and comparison using log-rank test were used. The Cox regression survival model was used to analyse the influence of the traits on the survival of the subjects. Statistically significant features were considered those with values of significance levels *p* < 0.05.

## 3. Results

### 3.1. Patient Characteristics According to the LPT

The study included 181 patients with KRAS wt mCRC. Concerning the LPT, patients were divided into two groups: 1. Patients with KRAS wt CRC localized in the right part of the colon (101 patients) and 2. Patients with KRAS wt CRC localized in the left part of the colon (80 patients). The average age of patients in relation to gender and the LPT is represented in Table 1. Male patients were statistically significantly older than female patients (men 62.8 ± 9.3; women 58.6 ± 11.7, *p* = 0.011). Men were significantly older than women in the group of patients with left localization (62.9 ± 9.1 vs. 57.6 ± 10.4, *p* = 0.015), while there was no significant age difference among genders in the group of patients with right localization of cancer (men 62.6 ± 9.8; women 59.9 ± 12.8, *p* = 0.227). There was no difference in the average age in relation to cancer localization (left 61.6 ± 9.6; right 61.4 ± 11.1, *p* = 0.902).

In male patients, the tumor was more often localized in the left part of the colon (60.5%), while in female patients it was more often localized in the right part of the colon (54.4%). The difference was at the limit of statistical significance (*p* = 0.061).

The distribution of patient characteristics by localization of primary colon tumor is shown in Table 2. The mucinous type of cancer was statistically significantly more represented in the group of patients with RCC (*p* = 0.001). Poorly differentiated tumor G3 was more common in patients with RCC, and G1 was more common in patients with LCC (*p* = 0.031). There was no statistically significant difference in the distribution of the disease stage concerning the localization of the primary tumor (*p* = 0.283) (Table 2). The average number of examined lymph nodes was statistically significantly higher (*p* = 0.003) in the group of patients with RCC (18.2 ± 9.6) than those with tumors on the left side (14.4 ± 8.7). The average number of organs/systems affected by metastases was statistically significantly higher (*p* = 0.035) in the group of patients with RCC (1.60 ± 0.79) than with LCC (1.37 ± 0.66) (Table 2).

LVI (*p* = 0.332), PNI (*p* = 0.905), and the occurrence of ileus (*p* = 0.737) did not show statistically significant differences with respect to the localization of the primary tumor (Table 2). The average disease-free survival (DFS) was 16.4 months (SD = 12.8; range 2–72). The most common metastases were found in the liver (72.9%), the lungs (21.5%), and the lymph nodes of the abdomen (18.2%) (Table 2). The DFS was 17.9 months (SD = 14.4; range 2–72) in the LCC group and 15.0 months (SD = 10.8; range 2–45) in the RCC group, with no statistically significant difference between the two groups (*p* = 0.243). In patients with RCC, metastases in the lymph nodes of the abdomen were statistically significantly more frequent (left—12.9%; right—25.0%; *p* = 0.036) (Table 2). Additionally, patients with RCC more commonly had metastases in the lymph nodes of the chest (left—1.0%; right—7.5%; *p* = 0.062), carcinosis of the peritoneum (left—3.0%; right—8.9%; *p* = 0.091), and metastases in the lymph nodes of the pelvis (left—5.0%; right—11.2%; *p* = 0.115) (Table 2).

In both groups of patients, the lymphocytic infiltrate was described as moderate (LCC—58.4% and RCC—51.2%) and there was no statistically significant difference in the distribution of findings on the presence of lymphocytic infiltrate in relation to the localization of the primary colon tumor (*p* = 0.176).

Anti-EGFR monoclonal antibodies (cetuximab, panitumumab) were commonly used as the third line of therapy, with 87.1% usage in left-sided localizations and 90.0% in right-sided localizations. There was no statistically significant difference in the localization of the primary tumor (*p* = 0.162) (Table 3).

### 3.2. Overall and Progression-Free Survival According to the LPT

**Figure 2:**
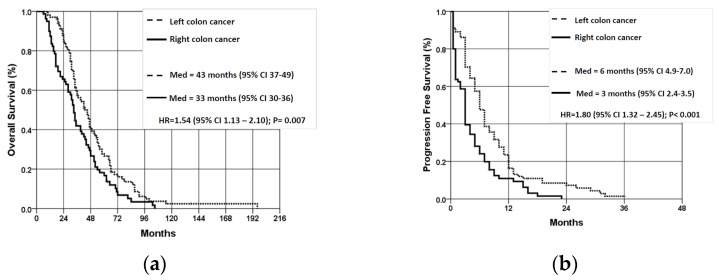
Overall survival (**a**) and progression-free survival (**b**) of patients with wt KRAS mCRC according to localization of the primary tumor. OS—overall survival; PFS—progression-free survival; LCC—left colon cancer; RCC—right colon cancer.

The average overall survival (OS) of patients with LCC was significantly better than that of patients with RCC (47.22 ± 27.9 vs. 38.2 ± 22.2 months, *p* = 0.007) (Figure 2a). In the stage III disease group, OS was better for patients with LCC (*n* = 39) compared to RCC patients (*n* = 35), but the difference was not statistically significant (*p* = 0.145). The average progression-free survival (PFS) for patients with LCC was significantly better than for patients with RCC (8.07 ± 7.17 vs. 4.27 ± 4.56 months, *p* < 0.001) (Figure 2b).

Regarding overall survival, we did not observe significant differences in OS of male vs. female patients in relation to the LPT (*p* = 0.461 for RCC, *p* = 0.212 for LCC). Moreover, there were no significant differences in PFS among male vs. female patients, according to the LPT (*p* = 0.177 for RCC, *p* = 0.953 for LCC).

Multivariate Cox regression analysis obtained independent predictors of cumulative survival (OS) of patients with colorectal cancer: mucinous adenocarcinoma (*p* < 0.001), right localization (*p* = 0.022), the presence of perineural invasion (*p* = 0.034) and the presence of tumors in resection margin (*p* = 0.049) (Table 4).

In the group of patients with LCC, Cox regression analysis obtained only one independent predictor of cumulative survival (OS), and that was the presence of perineural invasion (*p* = 0.038). Patients with perineural invasion had a 1.60 times lower survival rate than patients without perineural invasion (Table 5). In the group of patients with RCC, Cox regression analysis also obtained only one independent predictor of cumulative survival (OS), and that was mucinous adenocarcinoma (*p* = 0.038). Patients with mucinous adenocarcinoma had a 3.12 times lower survival rate (Table 5).

Cox regression analysis obtained independent predictors of the cumulative progression-free survival (PFS) of patients with colon cancer: mucinous adenocarcinoma (*p* = 0.001) and right localization (*p* = 0.004) (Table 6). Patients with mucinous adenocarcinoma had a 2.53 times lower survival rate than patients with classic NOS adenocarcinoma (Table 6). Patients with localization of the primary tumor on the right side of the colon have a 1.60 times lower survival rate than patients with the primary tumor localized on the left side of the colon (Table 6).

### 3.3. Response to the Anti-EGFR Therapy in KRAS Wild-Type mCRC Patients

According to the response to anti-EGFR therapy, patients with KRAS wild-type mCRC were divided into two groups: 1. Patients who responded well to anti-EGFR therapy (no disease progression after the first 3 months) (*n* = 102–56.4%) and 2. Patients who did not respond well to anti-EFGR therapy (with disease progression in the first 3 months) (*n* = 79–43.6%). Patients with LCC responded statistically significantly better to anti-EGFR therapy (*p* < 0.001) (left 70.3%: right 30.8%). Patients with mucinous adenocarcinoma responded statistically significantly worse to anti-EGFR therapy (*p* = 0.001) (mucinous 16.7%: NOS, Singnet ring c. 60.7%) (Table 7). There was no statistically significant difference in the distribution of responses to anti-EGFR therapy compared to other unfavorable prognosis parameters (Table 7).

### 3.4. HER2 Expression

**Figure 3:**
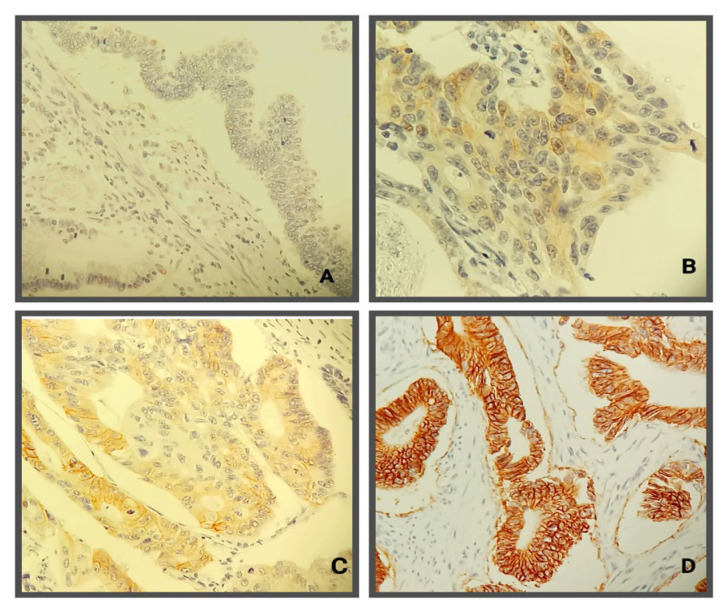
Immunohistochemical evaluation of HER2 antibody in colorectal cancer: (**A**)—negative tumor cells (Score 0); (**B**)—weak positivity of cellular membranes (Score 1+); (**C**)—incomplete membrane staining positivity in more than 10% of tumor cells in some tumor areas (Score 2+); (**D**)—complete membrane staining in more than 10% of colorectal tumor cells.

A HER2 positive (3+) score was registered in only 4 patients (2.21%), a potentially positive HER2 (2+) score in 8 patients (4.42%), a HER2 (1+) score in 26 patients (14.36%), and a negative score for HER2 overexpression in the other 144 patients (79.56%) (Figure 3). There was no statistically significant difference in the distributions of the unfavorable prognosis parameters and HER2 overexpression.However, among overall 38 HER2 positive patients, we noticed a significant difference in the distribution of HER2 positive scores according to the LPT (*p* = 0.038). HER2 (2+ and 3+) positive scores were more commonly detected among RCC patients than in LCC patients (62.5% and 75% vs. 37.5% and 25%, respectively), while HER2 (1+) score was more prevalent in LCC patients than in RCC patients (76.9% vs. 23.11%).

**Figure 4:**
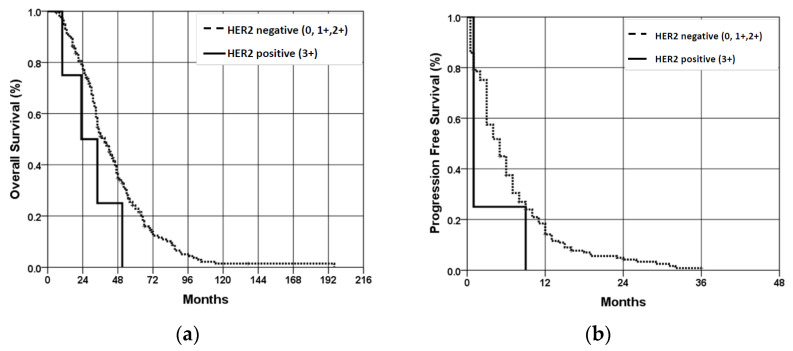
Overall survival (**a**) and progression-free survival (**b**) of patients with wt KRAS mCRC according to the HER2 expression status. OS—overall survival; PFS—progression-free survival.

OS of patients with HER2 positive (3+) scores is worse than that of patients with HER2 negative (0, 1+, 2+) scores; but, due to the small number of patients with HER2 positive scores, no statistically significant difference was obtained (*p* = 0.339) (Figure 4a). PFS of patients with HER2-positive results was statistically significantly worse (*p* < 0.001) compared to the patients with HER2-negative results (despite a small number of patients with HER2-positive results) (Figure 4b).

## 4. Discussion

The treatment strategy for mCRC is a significant aspect of clinical oncology due to the frequency of this disease. One-third of patients are diagnosed with the disease in the metastatic stage and half of them develop metastases during their illness, which significantly contributes to the high mortality rates associated with colorectal cancer [29]. According to our study data, at the time of diagnosis, the most common disease stage was stage IV (43.6%), followed by stage III (40.9%), and stage II was only 15.5%.

Standard chemotherapy, combined with targeted biological therapy, forms the foundation of treatment for patients with mCRC [30]. The principles of modern oncology aim to customize and personalize the treatment of patients with cancer. Tumor molecular characteristics, such as predictive biomarkers, are essential in tailoring and personalizing therapy. Prognostic and predictive factors are crucial in effectively treating mCRC patients, as they help identify those who are more likely to benefit from therapy. This approach helps minimize unnecessary exposure to toxicities and potential harm from therapy that is unlikely to be beneficial [31].

Multivariate analysis of 1,437,846 patients in 66 studies published between 1995 and 2016 showed that the location of the primary tumor in the distal (left) relative to the proximal (right) colon was associated with better survival (HR 0.82, CI95% 0.79–0.84, *p* < 0.001) [32]. In addition to outcomes, differences in epidemiology, pathogenesis, genetic and epigenetic changes, and molecular pathways between tumors localized in the left and right colons have also been identified [32]. Accordingly, the results of our study showed a connection between RCC and poorer prognosis in terms of shorter time to disease progression and overall survival compared to patients with LCC. However, our study did not analyse predictive markers, such as microsatellite instability (MSI) and BRAF status.

Tumor-infiltrating lymphocytes (TILs) reflect the host anti-cancer immune response [33]. Several studies analysed the TIL parameter as an independent prognostic and predictive factor, mainly in non-metastatic CRC in which a higher frequency of dense TILs in RCC was observed and low TIL is associated with worse prognosis. One of the reasons is MSI and tumors with deficient DNA mismatch repair (dMMR) which are more commonly located in the right colon [34]. In our study, in both groups of patients, the lymphocytic infiltrate was described as moderate (left—58.4% and right—51.2%) and there was no statistically significant difference in the distribution of findings on the presence of lymphocytic infiltrate in relation to the localization of the primary colon tumor (*p* = 0.176), which means that exact prognostic role of TILs in relation to the LPT needs further exploration, with consideration for the expression of other immune components of the tumor microenvironment.

The survival rates of patients with CRC in our study were 92.8% at 12 months and 22.3% at 5 years. The average survival time was 41.9 months (range 6–196) with a median survival of 37 months. The median cumulative survival (OS) for left-sided colon cancer was 43 months and for right-sided colon cancer was 33 months. Our study revealed that tumors on the right side were associated with poorer survival (HR 1.46; 95% CI 1.06–2.01; *p* = 0.022). Additionally, we found differences in survival by stages between tumors localized in the left and right parts of the colon. In the third stage, patients with LCC showed better overall survival compared to patients with RCC, but the difference was not statistically significant (*p* =0.145; LCC *n* = 39, RCC *n* = 35). Based on literature data [35], RCC is predominantly diagnosed at advanced stages of the disease, although we did not observe a statistically significant difference in the distribution of disease stages based on the tumor location (*p* = 0.283). Some studies suggest that patients with RCC have a better prognosis in the second and third stages compared to patients with LCC, while they have a worse prognosis in the fourth stage, including shorter progression-free survival and overall survival. This was observed in similar CRC studies, specifically in a study of 2027 patients treated with first-line chemotherapy, where RCC patients had better OS and PFS [8,36,37].

In stage III, patients with adjuvant therapy for RCC had a worse prognosis compared to those with LCC who received adjuvant chemotherapy [38]. Our study found that LCC generally also has a better prognosis in stage III. In the group of patients diagnosed in stage III with LCC (*n* = 39), overall survival was better compared to patients with RCC (*n* = 35) in the same stage, although the difference was not statistically significant (*p* = 0.145). Additionally, among patients in stage III who received adjuvant chemotherapy, those with LCC (*n* = 33) had better survival compared to those with RCC (*n* = 27), but the difference was not statistically significant (*p* = 0.234).

In a combined retrospective analysis, the predictive and prognostic value of primary tumor localization in the treatment of patients with anti-EGFR therapy, cetuximab, and panitumumab, was evaluated. The study involved 38% of the 5760 patients enrolled in the CRISTAL, FIRE-3, PEAK, PRIME, and CALGB studies [5,39]. The analysis revealed that primary tumor localization is an important prognostic factor and a significant predictive factor [5,39]. Therefore, when choosing the first-line systemic therapy for patients with mCRC one should take into consideration the molecular characteristics of the tumor as well as the localization of the primary tumor, in addition to patient preferences, comorbidities, and general condition of the patient [14,36]. Until a year ago, The Republic Health Insurance guidelines in our country approved the use of anti-EGFR therapy as the third line of treatment for patients with wild-type mCRC or as the first line of treatment for a limited number of cycles for potentially resectable metastasis in the liver. As a result, the majority of patients received anti-EGFR therapy as the third line of treatment (87.1% in the left and 90.0% in the right localizations). There was no statistically significant difference in relation to the localization of the primary tumor (*p* = 0.162). According to the National Comprehensive Cancer Network (NCCN) recommendations, the use of anti-EGFR therapy is suggested as the first line of treatment for patients with LCC. As the second and third lines, it is given regardless of the location of the primary tumor [30].

In our study, the PFS for metastatic colorectal cancer patients after 12 months of anti-EGFR monoclonal antibody treatment was 13.8%, and after 24 months, it was 4.1%. The average PFS was 6.39 months, with a median PFS of 5 months. Left-sided colon cancer patients had a better PFS compared to right-sided colon cancer patients (*p* < 0.001). The median cumulative progression-free survival for LCC patients was 6 months, while for RCC patients it was 3 months. RCC patients experienced a significantly shorter time to disease progression (PFS) (*p* = 0.004). Since the NRAS and BRAF status were not determined in this study, we cannot be certain about the factors influencing these results, which is one of the limitations of this study. It is evident that for our patients with RCC, the time to disease progression is shorter in the second and third line of systemic treatment.

One possible reason is the overexpression of the HER2 receptor in patients with mCRC. Unlike breast cancer, where about 30% of cases show HER2 overexpression, the data for CRC varies. Several studies suggest that only 3% of CRC patients have HER2 overexpression, with a higher prevalence in RAS/BRAF wild-type tumors at about 5–14% of cases [40,41,42,43], as they enhance HER2 amplification [36]. Though the overexpression of HER2 receptors in CRC is still relatively low, the role of HER2 expression itself is significant, as it impacts cancer development and progression leading to increased tumor cell proliferation and metastasis [44]. There are several factors that could explain the variation in expression rates, including small study populations, the use of different antibodies for immunohistochemistry (IHC), analysis of different patient subgroups with heterogeneous clinical and pathological characteristics of CRC, and the application of different scoring systems [41].

In our study, we observed a low percentage (2.21%—4 out of 181) of patients with mCRC that had overexpression of HER2 receptors. This low positivity may be due to several reasons. One of them can be the age of biological samples. Studies have analysed HER2 overexpression in relation to the location of the primary tumor. The PETACC-3 study found that HER2 receptor expression is more likely to be higher in tumors located on the left side of the colon [45]. Similar results were observed in patients with advanced colorectal cancer (CRC). A retrospective analysis revealed a higher incidence of HER2 overexpression in rectal cancer compared to descending or right colon cancer. In the HERACLES clinical study, among 33 patients with HER2-positive metastatic CRC, 64% had distal tumors and 21% had rectal tumors [42]. Retrospective data from the phase II EXPERT trial, which involved patients with high-risk, locally advanced rectal cancer who received neoadjuvant therapy with capecitabine and oxaliplatin with chemoradiotherapy with or without cetuximab, showed a 4.3% prevalence of HER2 overexpression. These findings are consistent with the 5.4% HER2-positivity rate for rectal cancer as described by Marshall and colleagues [46]. In our study, although the number of HER2-positive tumors was very small, we did not observe a higher incidence in the rectum.

The research findings on the relationship between HER2 receptor overexpression and overall survival in patients with colorectal cancer have been conflicting. Some studies show no difference in survival between patients with HER2 positive and negative CRC, while others suggest an association between HER2 receptor overexpression and poorer three-year (70.8% vs. 83.7%) and five-year survival (55.1% vs. 78.3%, *p* < 0.05) [47]. Our own study also found that the cumulative survival (OS) of patients with HER2 positive results is worse compared to patients with HER2 negative results. However, due to the small number of patients with HER2 positive results, we did not obtain a statistically significant difference (*p* = 0.339). The cumulative progression-free survival of patients with HER2-positive results was found to be statistically significantly worse (*p* < 0.001) compared to patients with HER2-negative tumors, despite the small number of patients with HER2-positive findings. With the low positivity of HER2 overexpression, more studies are now analyzing patients with low HER2 overexpression, such as tumors with HER2 (2+) positivity with a negative Fluorescence In Situ Hybridization test (FISH) finding or HER2 (1+). It has been demonstrated that patients with low HER2 overexpression have a better prognosis than HER2 positive and behave similarly to HER2 negative, standing out as a special subgroup of patients [45]. As our understanding of CRC and its genomic profile evolves, therapeutic strategies need to be updated to improve the survival of patients with this disease. Despite promising results from early research, targeted anti-HER2 therapy in the treatment of advanced CRC requires further research [48,49].

## 5. Conclusions

With this study, we wanted to highlight the importance of primary tumor location in the treatment of metastatic colorectal cancer. Our main treatment goal for these patients is to improve treatment effectiveness without increasing toxicity. By selecting the right patients for specific treatments, we can reduce potential therapy toxicity. Therefore, identifying the primary tumor location should be a standard prognostic and predictive factor in everyday clinical practice. We could not emphasize the prognostic and predictive significance of excessive HER2 expression due to the low percentage in our sample. Further randomized studies are necessary to establish the significance of HER2 receptor overexpression in mCRC patients. Our study had some limitations. We lacked data on comorbidities, which could impact overall survival. Additionally, we didn’t have all the molecular tumor characteristics such as NRAS and BRAF mutations, which also affect the response to anti-EGFR antibody therapy. Despite these limitations, our results reinforce the importance of primary tumor location as a crucial prognostic and predictive biomarker for everyday clinical practice.

## Figures and Tables

**Table 1:** Distribution of average age of the patients in relation to gender and the LPT.

Gender	Values	LCC *n* * = 75/26/101	RCC *n* ** = 49/31/80	Total *n* *** = 124/57/181	*p* Value ^1^
Male	average (SD)	62.9 (9.1)	62.6 (9.8)	62.8 (9.3)	0.833
	min–max	30–75	36–76	30–76	
Female	average (SD)	57.6 (10.4)	59.5 (12.8)	58.6 (11.7)	0.553
	min–max	37–79	21–76	21–79	
All	average (SD)	61.6 (9.6)	61.4 (11.1)	61.5 (10.3)	0.902
	min–max	30–79	21–76	21–79	
*p* value ^1^		0.015	0.227	0.011	

^1^ *p* values obtained using the Student’s *t*-test or the non-parametric Mann—Whitney test. LCC—left colon cancer; RCC—right colon cancer; SD—standard deviation, *n*—number of patients. * male, female, total number of patients for LCC. ** male, female, total number of patients for RCC. *** male, female, total number of patients.

**Table 2:** Pathohistological characteristics of CRC patients and the LPT.

Parameters	Values	LCC *n* = 101	RCC *n* = 81	*p* Value ^1^
Pathohistological type of tumor	Mucinous	3 (3.0%)	15 (18.8%)	**0.001**
	NOS	97 (96.0%)	63 (78.8%)	
	Singnet ring cell	1 (1.0%)	2 (2.5%)	
Differentiation	Good	15 (14.9%)	7 (7.8%)	**0.031**
	Moderate	75 (74.3%)	53 (66.2%)	
	Poor	11 (10.9%)	20 (25.0%)	
Resection margins	R0	85 (84.2%)	72 (90.0%)	0.442
	R1	6 (5.9%)	4 (5.0%)	
	Palliative surgery	10 (9.9%)	4 (5.0%)	
Tumor infiltration lymphocyte	Dense	19 (18.8%)	11 (13.8%)	0.176
	Poor	23 (22.8%)	28 (35.0%)	
	Moderate	59 (58.4%)	41 (51.2%)	
Stage at diagnosis	II	13 (12.9%)	15 (18.8%)	0.283
	III	39 (38.6%)	35 (43.8%)	
	IV	49 (48.5%)	30 (37.5%)	
No. of examined LN	Average (SD)	14.4 (8.7)	18.2 (9.6)	**0.003**
	Min–max	0–40	0–50	
No. of positive LN	Average (SD)	4.19 (4.7)	5.29 (5.8)	0.414
	Min–max	0–25	0–25	
No. of metastatic sites	Average (SD)	1.37 (0.66)	1.60 (0.79)	**0.035**
	Min–max	1–5	1–4	
Lymph node involvement	Present	6 (5.9%)	3 (3.8%)	0.742
Lymphovascular invasion	Present	76 (75.2%)	55 (68.8%)	0.332
Perineural invasion	Present	64 (63.4%)	50 (62.5%)	0.905
Ileus	Present	28 (27.7%)	24 (30.0%)	0.737
Metastatic sites	Liver	73.9%	72.5%	0.908
	Lung	20.8%	22.5%	0.781
	LN of abdomen	12.9%	25.0%	**0.036**
	Local recurrence	9.9%	6.2%	0.376
	LN of pelvis	5.0%	11.2%	0.115
	Carcinosis of peritoneum	3.0%	8.9%	0.091
	LN of thorax	1.0%	7.5%	0.062
	Ovary	3.0%	1.2%	0.785
	Bones	2.0%	1.2%	1.000
	Adrenal gland	2.0%	1.2%	1.000
	Brain	1.0%	1.2%	1.000
	Urinary bladder	2.0%	0.0%	0.582
	Prostate	1.0%	0.0%	1.000
	Pancreas	1.0%	0.0%	1.000

^1^ All *p* values obtained using the χ^2^ test, except for number of examined LN, positive LN, and metastatic sites where Student’s *t*-test or the non-parametric Mann—Whitney test was used. LCC—left colon cancer; RCC—right colon cancer; NOS—not otherwise specified; LN—lymph nodes; SD—standard deviation.

**Table 3:** Systemic treatment wt mCRC with anti-EFGR therapy according to the LPT.

Line of Therapy	LCC *n* = 101	RCC *n* = 80	Total *n* = 181	*p* Value ^1^
II	3 (3.0%)	5 (6.2%)	8 (4.4%)	0.162
III	88 (87.1%)	72 (90.0%)	160 (88.4%)	
IV	10 (9.9%)	3 (3.8%)	13 (7.2%)	

^1^ *p* value obtained using the χ*2* test. LCC—left colon cancer; RCC—right colon cancer.

**Table 4:** Unfavorable prognosis parameters for OS in CRC patients.

Predictors in CRC	B	SE	*p* ^1^	Exp(B)	95% CI
Mucinous adenocarcinoma	0.990	0.269	**<0.001**	2.69	1.59–4.56
Right localization of primary tumor	0.375	0.164	**0.022**	1.46	1.06–2.01
Perineural invasion	0.355	0.167	**0.034**	1.43	1.03–1.98
Resection margins end palliative surgery	0.518	0.263	**0.049**	1.68	1.02–2.81

^1^ *p* value obtained using the Cox regression analysis (conditional forward method).

**Table 5:** Unfavorable prognosis parameters for cumulative OS in patients with LCC and RCC.

Predictors in LCC	B	SE	*p* ^1^	Exp(B)	95% CI
Perineural invasion	0.468	0.225	**0.038**	1.60	1.03–2.48
Predictors in RCC	B	SE	*p* ^1^	Exp(B)	95% CI
Mucinous adenocarcinoma	1.136	0.304	**<0.001**	3.12	1.72–5.66

^1^ *p* value obtained using the Cox regression analysis (conditional forward method).

**Table 6:** Unfavorable prognosis parameters for PFS in CRC patients.

Predictors in CRC	B	SE	*p* ^1^	Exp(B)	95% CI
Mucinous adenocarcinoma	0.927	0.270	**0.001**	2.53	1.49–4.29
Right localization of primary tumor	0.471	0.165	**0.004**	1.60	1.16–2.21

^1^ *p* value obtained using the Cox regression analysis (conditional forward method).

**Table 7:** Unfavorable prognosis parameters according to response to anti-EGFR therapy.

Parameters	Characteristics	Not Responded *n* = 79	Good Response *n* = 102	*p* Value ^1^
LPT	Right	49 (61.2%)	31 (30.8%)	**<0.001**
	Left	30 (29.7%)	71 (70.3%)	
Pathohistological type of tumor	NOS, SRCC	64 (39.3%)	99 (60.7%)	**0.001**
	Mucinous	15 (83.3%)	3 (16.7%)	
Differentiation	Good	7 (31.8%)	15 (68.2%)	0.233
	Moderate, poor	72 (45.3%)	87 (54.7%)	
Stage of disease	II	11 (39.3%)	17 (60.7%)	0.613
	III, IV	68 (44.4%)	85 (55.6%)	
No. examined LN	<11	18 (34.6%)	34 (65.4%)	0.120
	>12	61 (47.3%)	68 (52.7%)	
Involved LN	No	16 (36.4%)	28 (63.6%)	0.263
	Yes	63 (46.0%)	74 (54.0%)	
Resection margins and palliative surgery	R0	69 (43.9%)	88 (56.1%)	0.541
	R1, R2	10 (41.7%)	14 (58.3%)	
LVI	No	20 (40.0%)	30 (60.0%)	0.188
	Yes	59 (45.0%)	72 (55.0%)	
PNI	No	25 (37.3%)	42 (62.7%)	0.834
	Yes	54 (47.4%)	60 (52.6%)	
TIL	Poor, moderate	25 (49.0%)	26 (51.0%)	0.361
	Dense	54 (41.5%)	76 (58.5%)	

^1^ *p* value obtained using the χ*^2^* test. LPT—location of the primary tumor; NOS—not otherwise specified; SRCC—Signet ring cell carcinoma; LN—lymph nodes; LVI—lymphovascular invasion; PNI—perineural invasion; TIL—tumor infiltration lymphocyte.

## Data Availability

The raw data supporting the conclusions of this article will be made available by the authors on request.
